# Lanthanide-Doped Upconversion Luminescent Nanoparticles—Evolving Role in Bioimaging, Biosensing, and Drug Delivery

**DOI:** 10.3390/ma15072374

**Published:** 2022-03-23

**Authors:** Palak Jethva, Munira Momin, Tabassum Khan, Abdelwahab Omri

**Affiliations:** 1 SVKM’s Dr. Bhanuben Nanavati College of Pharmacy, Mumbai 400 056, India; palakjethva00@gmail.com; 2 Department of Pharmaceutics, SVKM’s Dr. Bhanuben Nanavati College of Pharmacy, Mumbai 400 056, India; munira.momin@bncp.ac.in; 3 Department of Pharmaceutical Chemistry, SVKM’s Dr. Bhanuben Nanavati College of Pharmacy, Mumbai 400 056, India; 4 The Novel Drug & Vaccine Delivery Systems Facility, Department of Chemistry and Biochemistry, Laurentian University, Sudbury, ON P3E2C6, Canada

**Keywords:** upconversion nanoparticles, luminescence, lanthanides, biosensors, bioimaging

## Abstract

Upconverting luminescent nanoparticles (UCNPs) are “new generation fluorophores” with an evolving landscape of applications in diverse industries, especially life sciences and healthcare. The anti-Stokes emission accompanied by long luminescence lifetimes, multiple absorptions, emission bands, and good photostability, enables background-free and multiplexed detection in deep tissues for enhanced imaging contrast. Their properties such as high color purity, high resistance to photobleaching, less photodamage to biological samples, attractive physical and chemical stability, and low toxicity are affected by the chemical composition; nanoparticle crystal structure, size, shape and the route; reagents; and procedure used in their synthesis. A wide range of hosts and lanthanide ion (Ln^3+^) types have been used to control the luminescent properties of nanosystems. By modification of these properties, the performance of UCNPs can be designed for anticipated end-use applications such as photodynamic therapy (PDT), high-resolution displays, bioimaging, biosensors, and drug delivery. The application landscape of inorganic nanomaterials in biological environments can be expanded by bridging the gap between nanoparticles and biomolecules via surface modifications and appropriate functionalization. This review highlights the synthesis, surface modification, and biomedical applications of UCNPs, such as bioimaging and drug delivery, and presents the scope and future perspective on Ln-doped UCNPs in biomedical applications.

## 1. Introduction

**Figure 1:**
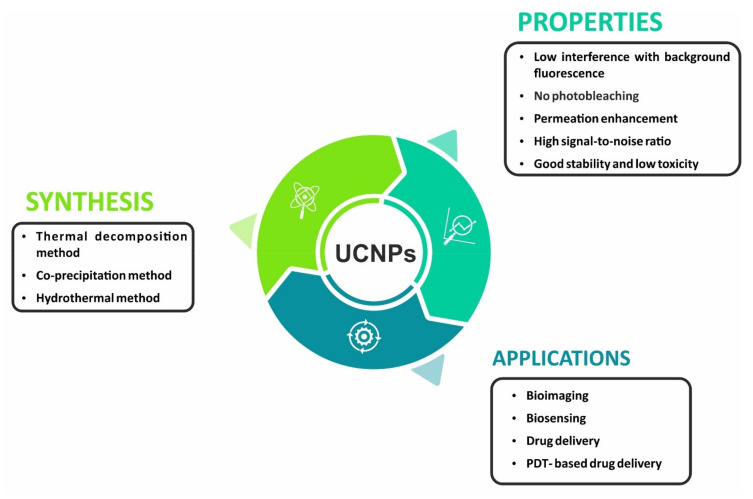
Synthesis, properties, and applications of UCNPs.

Nanotechnology has ushered in a paradigm shift following recent breakthroughs and recognition as one of the most crucial areas of upcoming technology [1]. In layman’s terms, it is a method of manipulating at the atomic/molecular level with materials so small that nothing can be constructed any smaller for its utilization in the design, characterization, synthesis, and application of materials, structures, and devices. It has applications in a variety of systems, including physical, chemical, and biological systems with diameters ranging from atoms to submicrons [2]. Nanoparticles (NPs) have always existed in nature, mostly in the form of dust and smoke with a diameter ranging from 1 to 100 nanometers [3]. They have a wide range of applications in life sciences from basic biophysical studies to clinical therapies. NPs have a high specific surface area that confers high binding capability and distinctive optical features [4]. Usually, when light emission occurs, the wavelength of emitted light is longer than that of the excitation light, implying that the emitted photon energy is lower than the absorbed one. However, this phenomenon can be reversed in some cases, i.e., the emission wavelength is shorter than that of the incident light. This distinct nonlinear anti-Stokes phenomenon occurs via a process called as upconversion. Furthermore, when a nanoparticle absorbs energy, a portion of that energy can be converted into a form of electromagnetic radiation with energies greater than thermal radiation, resulting in luminescence. Upconverting nanoparticles with luminescent properties, also known as upconverting luminescent nanoparticles (UCLNPs), have piqued scientific interest as they have a unique nonlinear optical feature wherein two or more photons are sequentially absorbed and ultraviolet/visible/near-infrared light is emitted at a wavelength shorter than the excitation wavelength [5,6]. Figure 1 depicts the synthetic procedures of UCNPs along with their properties and potential application in biomedical fields.

**Figure 2:**
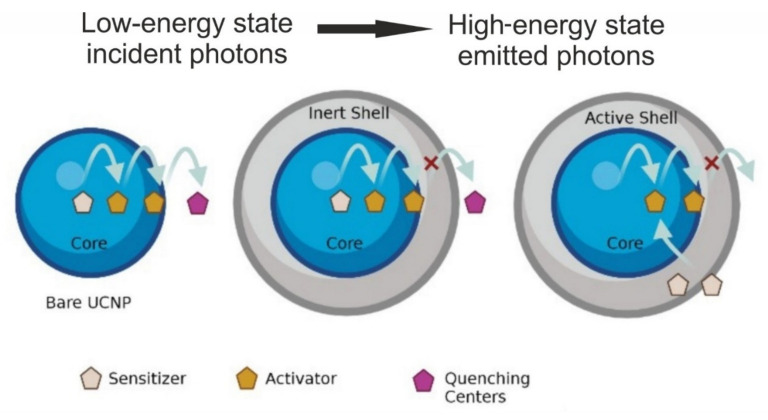
Structure of core–shell upconversion nanoparticle.

The upconversion luminescence (UCL) mechanism takes place due to interaction between the low-energy incident photons and the long-lived intermediate state of the luminescent entity. Excited-state absorption (ESA), photon avalanche (PA), and energy transfer upconversion (ETU) are the three primary mechanisms of UCL. The material is excited to a higher energy level via energy transfer, excited-state absorption, and triplet-triplet annihilation, and subsequently emits a high-energy photon [7]. Upconversion efficiency can be achieved by co-doping sensitizer ions along with activator ions having a closely matched intermediate-excited state [8,9,10]. The doping process involves a rational design that offers optimal interactions of a network of sensitizer and activator ions. (Figure 2). The inert shell does not contain the sensitizer or activator ion; hence, the shell layer removes the energy transfer route from the activator (or sensitizer) to the surface-quenching centers and reduces the possibility of nonradiative transition translating to increased upconversion efficiency. The upconversion efficiency of the NPs depends on the distance between the dopants and this makes the doping concentration, a deciding factor in maximizing the energy transfer process and consequently the luminescence performance of the NP [11,12].

The properties of UCNPs can be modulated using suitable host–dopants combinations, core–shell nanostructures, and energy exchange of nanostructures with “alien species” (such as organic dyes, quantum dots) [13]. These attractive features make UCNPs an excellent biomaterial for multimodal tumor imaging, drug delivery, cell labeling, sensing, PDT, and photothermal therapy (PTT) [14,15,16].

## 2. Properties and Composition of UCNPs

UCNPs possess several advantages including (i) absence of autofluorescence, resulting in enhanced signal-to-noise ratio and higher detection sensitivity; (ii) deeper biological tissue near-infrared (NIR) light penetration, causing less photodamage; and (iii) low-power NIR-laser-based excitation. The UCNP signal can be quantified because there is a direct correlation between the number of particles and the signal strength. Furthermore, there is extensive scope for multiplexing in UCNPs because the emission spectrum of the signal is very narrow and one can have any number of colors in one image. The additional features of UCNPs include narrow emission peaks, multiplexing, better chemical and physical stability, low toxicity, and no photobleaching. 

**Figure 3:**
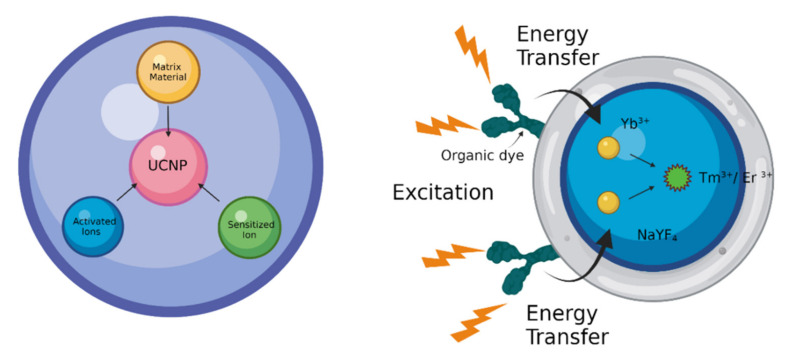
Components of UCNPs and mechanism of energy transfer in UCNPs.

UCNPs consist of inorganic host molecules with a rare earth (RE)-based lanthanide/actinide dopant incorporated in the host’s lattice. Photon upconversion is reported for a variety of dopants embedded in suitable host molecules, for example, solids doped with transition metal ions such as Ti^2+^, Ni^2+^, Mo^3+^, Re^4+^, or Os^4+^. Lanthanide-doped (Ln^3+^) materials have the highest upconversion efficiencies at room temperature. Luminescence is highly dependent on the transition of electrons in the 4f subshell. Lanthanides are metal ions having their 4f energy level filled and the valence electrons are shielded from external interactions. While all lanthanides (from lanthanum to lutetium) undergo upconversion, only erbium (III), holmium (III), and thulium (III) can absorb and advance to the specified levels of the visible and UV ranges because their inner shell electrons are shielded by the 5s 5p subshells, resulting in a large number of metastable energy states, making them amenable to upconversion (Figure 3). Even though a single lanthanide ion can induce the upconversion (UC) effect, co-doping is normally preferred to enhance the UC efficiency, since most lanthanide ions have low absorption cross-sections, resulting in weak emission [17]. The absorption can be increased by raising the dopant concentration of lanthanide ions per single nanocrystal. However, high doping concentrations result in a phenomenon called concentration quenching, which limits the quantity of dopant used. A high amount can lead to radiation-free deactivation and cross-relaxation processes, hence must be kept below 2 mol % to avoid loss of excitation energy. It is necessary to co-dope sensitizer ions alongside activator ions with a closely matched intermediate-excited state to achieve high upconversion efficiency. These highly absorbing sensitizer ions provide efficient nonradiative energy transfer to activator ions and result in improved absorption [18,19]; Yb^3+^ ions are the most often utilized sensitizers for Er^3+^, Tm^3+^, or Ho^3+^ doped UCNPs [20]. A few examples of nanoparticles with upconversion luminescence include NaYF_4_:Yb:Er [21,22], Y_2_O_3_:Yb:Er [23], Gd_2_O_2_S:Eu^3+^ [24,25], NaPrF_4_:Yb:Tm [26], and Cs_3_Lu_2_Br_9_:Er^3+^ [27]. Of these, Ln-doped UCNPs, NaYF_4_ co-doped with Yb^3+^/Er^3+^ or Yb^3+^/Tm^3+^ nanoparticles demonstrated the highest UC efficiency with usage in cellular and in vivo animal imaging [28].

## 3. Synthesis of UCNPs

Achieving high luminescent efficiency of UCNPs is a major goal; therefore, synthesizing UCNPs using appropriate techniques is essential to obtain UCNPs with well-defined size, shape, content, and phase. UCNPs are reported to be generally synthesized by three methods: thermal decomposition, co-precipitation, and hydrothermal synthesis. 

### 3.1. Thermal Decomposition Method

**Figure 4:**
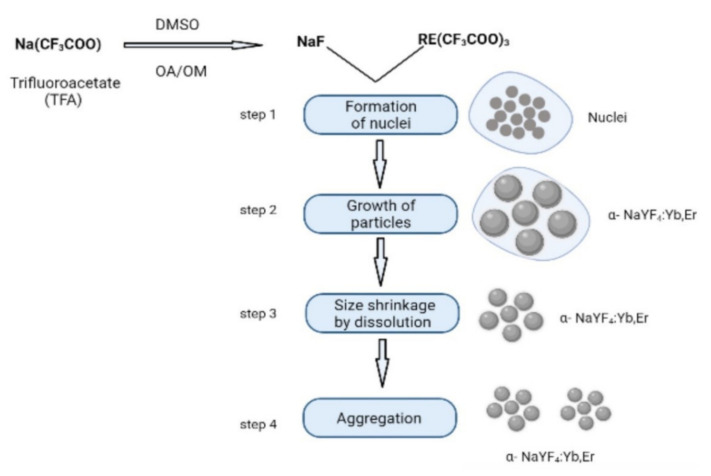
Synthesis of α-NaYF_4_: Yb^3+^/Tm^3+^ or α-NaYF_4_: Yb^3+^/Er^3+^ UCNPs via thermal decomposition method.

Thermal decomposition method is based on the traditional solvent thermal method, and includes addition of trifluoroacetic acid RE salt and RE halide to the reaction system, which decomposes at high temperatures. This method is used to produce phase-pure single-crystalline UCNPs of uniform size within short time duration. Different sizes and shapes of NaYF_4_: Yb^3+^/Tm^3+^ or YB^3+^/Er^3+^ UCNPs can be generated by adjusting reaction time, reaction temperature, and reagent concentration. When a trifluoroacetate, such as Na (CF_3_COO), is dissolved in high-boiling organic solvents such as dimethylformamide (DMF) and dimethyl sulfoxide (DMSO) with the help of surfactants having polar capping groups and long hydrocarbon chains, such as oleic acid (OA), and omeylamine (OM), NaF is formed [29]. A rare-earth-doped metal trifluoroacetate is added to NaF, resulting in formation of α-NaYF_4_: Yb^3+/^Tm^3+^ or α-NaYF_4_: Yb^3+^/Er^3+^ UCNPs [30]. The thermal decomposition method comprises four steps that are depicted in Figure 4 [31]. It is essential to maintain appropriate reaction conditions (high temperature and pressure, capping ligand, heating and cooling rates, reaction duration, solvent and reagent concentrations) as UCNPs are sensitive to oxygen impurities and are anhydrous, requiring long reaction time and higher reaction temperatures for synthesis to yield high-quality monodispersed nanoparticles of desired nanocrystal morphology and size. OA and OM (capping ligands) bind to the surface of NPs via outward hydrophobic alkyl chains, making it hydrophobic. This method releases toxic fluorinated and oxyfluorinated carbon species into the environment. The synthetic particles are usually oil-soluble and have a high toxicity. Surface modification strategies are used to improve the water solubility, biocompatibility, and reduce the toxicity of UCNPs; their functionalization also expands the scope of their application. Thermal decomposition is the most common method used for synthesis high-quality UCNPs, in spite of some limitations pertaining to its industrial application. In most cases, the synthesis process requires the use of high temperatures, expensive and air-sensitive precursors and solvents, and is accompanied by the generation of toxic byproducts.

### 3.2. Co-Precipitation Method

The co-precipitation method is a simple cost-effective operational process, requires mild temperature conditions, produces fewer harmful byproducts, and provides a better solution from an industrial translational perspective, offering more environmentally friendly reagents containing inorganic RE salts. This approach is used to make ultrasmall Ln-UCNPs (2–10 nm) with improved crystallinity and luminescence efficiency [32]. Firstly, the formation of tiny amorphous Ln-UCNPs is controlled by a coordinating ligand at room temperature. Polyvinylpyrrolidone (PVP) may be used as the surface-coordinating ligand. The temperature is then increased to induce particle growth, which leads to the formation of nanocrystals via the Ostwald ripening mechanism [33]. Chen et al. constructed a dye-sensitized core–shell NaGdF_4_: Yb, Er@NaGdF_4_:Yb, Nd UCNPs to detect H_2_S for its application in cell and living body research via the co-precipitation method [34]. Liu et al. proposed a two-step reaction methodology for the solvothermal co-precipitation method to synthesize ultrasmall core–shell UCNPs, which resulted in a strong luminescence as a prototype for preparing UCNPs with high efficiency [35]. Lei et al. synthesized NaBiF_4_:Yb^3+^/Er^3+^ under room temperature via a simple hydrothermal method combined with a succedent calcining process. The substitution of yttrium with bismuth species considerably reduced the reaction conditions while maintaining upconversion luminescence, making them a potential candidate for lighting and solar cell applications [36].

Yi et al. synthesized α-NaYF_4_: Yb, Er UCNPs via a homogeneous nucleation process by injecting a RE-EDTA complex into NaF solution with vigorous stirring. The resulting UC fluorescent intensity of the UCNP was too low for biological labeling. Hence, an annealing treatment was given to enhance the UC fluorescent intensity [37]. Annealing of capping reagents results in carbonization and the hydrophilicity of the NPs is decreased. Hence, surface modification is required to improve the hydrophilicity of these NPs and to allow further increase in the size of NPs. This limits the utility of the co-precipitation method for the synthesis of RE-doped NaYF_4_ UCNPs for biological applications. 

### 3.3. Hydrothermal Method

The hydrothermal method is usually carried out in a special closed reactor under high temperature and high pressure with water or organic solvents, and produces fewer harmful byproducts. In this method, a chemical reaction occurs when positive ions and negative ions are exposed to temperatures and pressures above their critical points in polar liquids, resulting in the formation of UCNPs. Zhang et al. used the hydrothermal method for the synthesis of Yb^3+^ and Er^3+^ co-doped NaYF_4_ upconversion luminescent materials and Ag nanoparticles coated with SiO_2_, which enhances the luminous intensity and has low cytotoxicity because of the SiO_2_ coating [38]. Nampi et al. synthesized single-crystalline, stable, aqueous Yb^3+^/Er^3+^doped BaYF_5_ UCNPs with polyethyleneimine (PEI) via hydrothermal route. With appropriate surface modifications, these particles could be adopted for biosensing of disease markers and bioimaging applications [39]. Heer et al. described a solution-based method for increasing the solubility of lanthanide precursors in supercritical polar solvents that favors the nanoparticles’ development rate. Surfactants are also added in the formulation process as growth control agents for nanocrystals [40]. The nucleation and growth stages are separated by the creation of an Ln^3+^ surfactant complex. The particle size distribution and morphology may be fine-tuned by adjusting the surfactant/Ln^3+^ ratio [29]. After the nanoparticles’ development stage is completed, the surfactant serves to cap ligands, limiting further aggregation [41]. Yi et al. synthesized UCNPs using the hydrothermal method and the results show that Ni^2+^ doping increases the UCL intensity, resulting in a change in morphology (hexagons to nanorods) with increasing size [42]. However, the disadvantage of this method is that the resulting UCNPs usually have a wider particle size distribution, and byproduct remnants can occasionally attach to the surface of UCNPs, making removal difficult. It should be highlighted that the hydrothermal preparation method’s future industrialization is still hindered by the long reaction time, which can range from 12 to 24 h or even longer. Apart from that, high pressures, large solvent volumes, and poor reproducibility are all major concerns that must be addressed.

In addition to these synthetic routes, ionothermal, microwave-assisted heating, sol-gel, microemulsion method, and combustion are reported for the synthesis of Ln-doped UCNPs. Microwave-assisted heating, for example, is a one-step process that takes much less time and energy than conventional methods and is regarded as a green method for synthesizing nanocrystals [43]. The combustion and microemulsion processes are utilized less commonly due to various intrinsic limitations such as difficulty to control the size and agglomeration, and poor light performance. Table 1 lists the methods used in the synthesis of UCNPs, along with their advantages and disadvantages.

## 4. Surface Modification of UCNPs

**Figure 5:**
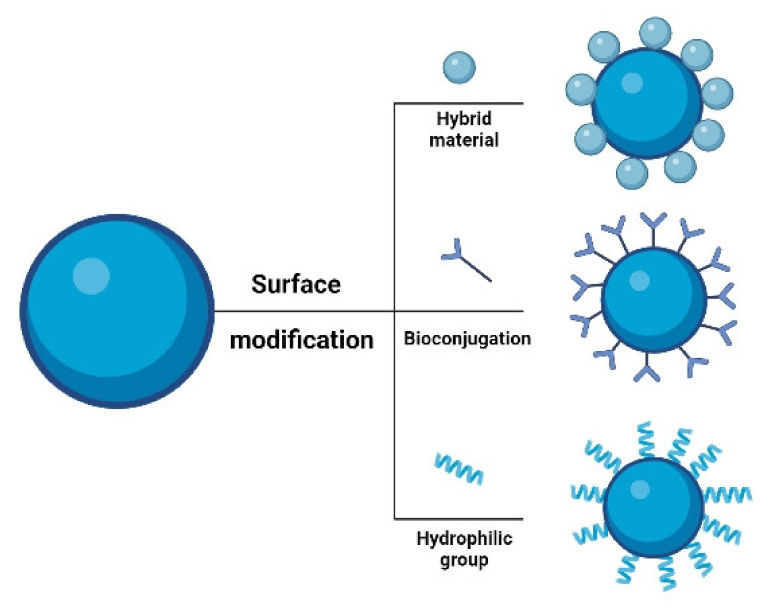
Surface modification of UCNPs for modulating its properties.

Using a combination of energy migration and core–shell structural engineering to improve properties for a wide range of activators would widen the range of applications for lanthanide-doped nanoparticles [53,54,55]. Surface modification is required for specific nanomaterials to fulfil various biological activities, in biosciences such as disease therapy (particularly cancer) [56,57], detection [58], and immunoassay [59]. Understanding surface functionalization of Ln-doped UCNPs is critical for improving UC efficiency and aqueous solubility [60]. UCNPs are typically hydrophobic; hence, creating water-soluble Ln-doped UCNPs is critical for biological applications. Hydrophilic ligands coat the surface of UCNPs and can be dispersed in nonpolar organic solvents such as hexane and heptane. They lack functional groups for conjugation with biomolecules, such as carboxyl or amino groups. On the other hand, UCNPs capped with hydrophobic surfactants are not biocompatible and cannot be used immediately as they do not disperse in water. The surface characteristics of UCNPs determine their biocompatibility in in vitro and in vivo [61]. As a result, surface modification with an inorganic shell layer and an organic capping ligand is favored to overcome these difficulties. The surface of the NP can be coated by selection of an appropriate polymer or surfactant such as polyethyleneglycol (PEG), polyethylenimine (PEI), polyvinylalcohol (PVA), carboxydextran, or oleic acid (OA). Ligand removal, ligand oxidation, layer-by-layer deposition, acid treatment, and ligand exchange are some of the methods used to alter the surface properties of UCNPs, conferring high hydrophilic attributes [62,63]. Several surface modifications and depicted in Figure 5. Kostiv et al. synthesized uniformly sized NaYF_4_:Yb^3+^/Tm^3+^@NaYF_4_-PEG-Alk nanoparticles and bioconjugated it with a click reaction of pAbF-azide or SA azide for its bioanalytical applications, such as immunoassays [64]. Wang et al. [65] used a robust ligand exchange technique to develop a novel method for converting hydrophobic inorganic UCNPs to hydrophilic UCNPs. They were synthesized using the hydrothermal method and converted into carboxyl-modified UCNPs by replacing the original capping OA ligands on the surface of nanocrystals with PAA in a diethylene glycol (DEG) solvent at a high reaction temperature, rendering water-soluble nanocrystals.

## 5. Applications of UCNPs

UCNPs are reported to be used across a diverse spectrum of biological applications such as bioimaging, therapeutics, drug delivery, and biosensing. Discussion of these applications is elaborated in this section.

### 5.1. UCNPs in Bioimaging

**Figure 6:**
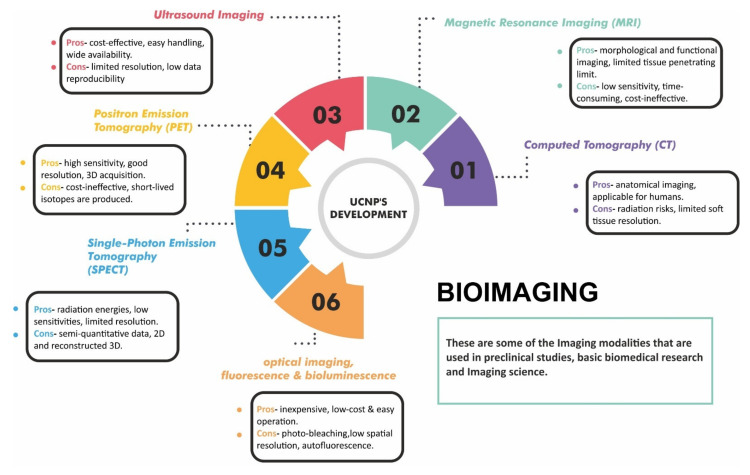
Development of UCNPs in imaging modalities.

Life science and nanomedicine have advanced at an incredible rate to improve life quality, which has encouraged significant research in the fields of bioimaging and technology. Bioimaging is a noninvasive technique for visualizing biological processes, allowing observation of subcellular structures, cells, tissues, and even complete multicellular creatures. UCNPs have versatility in generating nanoplatforms that have imaging and therapeutic modalities [66,67]. In addition, they have potential biomedical applications in early-stage diagnosis and monitoring therapy intervention, and have emerged as a novel carrier for small animal imaging, including tumor-targeted imaging, lymphatic imaging, and vascular imaging [68]. As UCNPs use a two-photon absorption mechanism, their energy emission and upconversion efficiency are higher when compared to other traditional technologies involving organic dyes and quantum dots. Hence, they have demonstrated promising results in optical-imaging-guided drug delivery and have become exclusive candidates in the field of bioimaging (in vivo and in vitro imaging of animal tissues) due to their unique photophysical properties such as lack of autofluorescence and deep-tissue-reaching resulting from luminescence after NIR excitation, resistance to photobleaching, and blinking [69]. In vitro cellular imaging involves targeting Ln-doped UCNPs to a subcellular component such as a membrane protein. The enhanced cellular uptake efficiency of positively charged UCNPs results in brighter in vitro cellular imaging. UCNPs can be employed to improve image contrast and sensitivity of in vivo imaging [70,71]. However, more research and testing are needed to fully comprehend the effect of nanoparticle size on optical properties to help optimize them for in vitro luminescence imaging [72,73,74]. Various imaging modalities used in pre-clinical studies and imaging science are depicted in Figure 6. Of these imaging modalities, PET/MRI shows the greatest clinical potential because MRI uses protons present in the soft tissue in the human body contrast paired with PET sensitivity. MRI produces better in vivo images together with good deep-tissue contrast and spatial resolution because tissue penetration is limited to a few mm [75].

Tian et al. synthesized a novel Nd^3+^-sensitized Er@Y@Nd@Gd core@multishell UCNP with carboxy-terminated silica and UEA-I. This UCNP@SiO_2_-UEA-I has high SW480 tumor-targeting potential. The ultrasmall SW480 tumor in the Balb/c nude mouse is observed using in vivo UCL imaging with UCNP@ SiO_2_-UEA-I. The findings suggest that the red UCL-emitted UCNP@SiO_2_-COOH bioconjugates with a minimized heating effect have much potential for sensitive deep-tissue biomedical imaging; synthesized UCNP@SiO_2_-UEA-I can serve as an efficient optical probe for early diagnosis of SW480 tumors [76]. A multifunctional nanocluster bomb (UCGM nanoparticles), CeOx, graphite-C_3_N_4_ (g-C_3_N_4_) NPs, and metformin (Met) were developed to alleviate hypoxia by oxidizing H_2_O_2_ to O_2_. In vivo UCL was used to monitor the distribution of UCGM NPs after they were injected into HepG_2_ tumor-bearing mice. Meanwhile, g-C_3_N_4_ NPs were released from UCGM NPs and, due to their tiny size, they penetrate tumor tissue deeply. Before the IV injection of UCGM NPs, there was no in vivo reinforcement in CT in the tumor, but after the injection, there was a significant CT signal in the tumor. UCGM NPs can serve as multifunctional theranostic agent for use in PTT/PDT-based therapy guided by UCL/CT/MRI trimodel imaging in vivo because of their great capacity to combat tumor hypoxia [77].

NaYF_4_ is conventionally the most notable system that has been employed in cellular and in vivo imaging. Hence, small-animal imaging and deep-tissue imaging are performed using NaYF_4_ UCNPs doped with Er^3+^ or Tm^3+^ that emit 800 nm NIR light, and have a better contrast that can be further improved by separating the long-lived luminescence from scattered light by tissue components using time-gated measurements [78]. In addition, there is significant use of UCNPs for super-resolution imaging based on stimulated emission depletion microscopy (STED), as reported by the Kolesov et al. [79]. The development of UCNP probes has promoted the translation of UCNPs application in cellular imaging. It is believed that UCNPs comprising both opportunities and limitations will attract great concern as probes for super-resolution microscopy [80]. Table 2 summarizes recent studies on the development of UCNPs for bioimaging, together with their composition/modifier and the synthesis route used to create UCNPs.

### 5.2. UCNPs in Biosensing 

**Figure 7:**
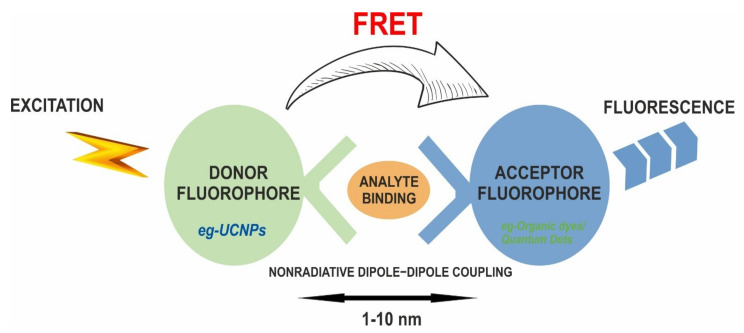
Mechanism of FRET resulting in fluorescence.

Many optically based biosensing methods have great potential for monitoring biomedical substances at clinically relevant levels; however, many of these methods encounter the problem of serum or whole blood autofluorescence and upconversion materials help resolve this issue. UCNPs exhibit minimal autofluorescence and deep tissue penetration, allowing them to be used in biological and environmental monitoring, detection, and sensing. Once UCNPs are synthesized, they can be easily functionalized and utilized as sensing nanoprobes to detect circulating cancer biomarkers. The capabilities of Ln-doped UCNPs in various biological sensing/detection depend on resonance energy transfer (RET) [88]. Fluorescence resonance energy transfer (FRET) is a nonradioactive process that describes the transfer of energy from a donor fluorophore to an acceptor fluorophore via a nonradiative dipole−dipole coupling [89,90] (Figure 7). These FRET systems were created by combining UCNP’s as an energy donor and organic dyes or QDs that act as an energy acceptor [91]. FRET typically offers greater freedom for upconverted emission wavelengths than the one which is formed solely by the Ln^3+^ ions. The FRET systems, which were developed using upconversion nanoparticles and gold nanoparticles for the detection system, have significant implications for biosensing and are frequently used in UCNP-based homogeneous tests. Zhen et al. proposed the use of UCNPs with confined emitters and bared surfaces as the luminophore and Ca^2+^ as a proof-of-concept target to develop a LRET-based nanoprobe. By simply altering the Ca^2+^ receptor into different recognition units, such as peptides, aptamers, and small-molecule ligands, this technique can be adapted to build numerous UC nanoprobes [92]. Their theory proposed the sandwich structure upconversion nanoparticles (SWUCNPs) with a core–inner-shell–outer-shell architecture, wherein the emitting ions (Ln^3+^) are precisely placed in the inner shell near the particle surface, close to external energy acceptors.

Several research groups have reported the usage of Ln doped-UCNPs in FRET-based detection [93]. Nd^3+^-UCNPs sensor is an excellent emitter that has low autofluorescence and a high penetration depth to biological samples. The oleic ligands from the core@shell UCNPs were readily removed by acid treatment, resulting in water-dispersible Nd^3+^-UCNPs. An ultrasensitive and selective approach for detecting miRNA with surface functionalized Au NPs-thiolated single-stranded DNA was proposed based on the chiroplasmonic and upconversion luminescence features of Au-UCNP pyramids as intracellular nanoprobes. The results indicate that an ultrasensitive and efficient chiral nanostructure-based detection approach can be used to identify biological systems [94]. Another intriguing aspect of UCNPs is their luminescence intensity, which is substantially temperature dependent. The nanoparticle-based thermometer provides a variety of alternatives for measuring the two-dimensional distribution of temperature and is important for understanding subcellular processes [95]. Lin et al. created a model of a multilayer nanocomposite structure NaYbF_4_:2%Er@NaYF_4_@MSN@Au@SiO_2_@Ag_2_S to depict the temperature distribution among nanoparticles. They concluded that these temperature-sensitive luminous probes, which are nanoscale in size, can be useful tools for high-resolution thermal sensing in micro areas [96]. Wolfbeis et al. studied temperature sensing with UCNPs of various sizes and RE dopants and found that the core–shell structured hexagonal (NaYF_4_:Yb20%Er2%)/NaYF_4_ UCNPs are better suited for temperature sensing because they can resolve temperature differences of less than 0.5 °C in the physiological range between 20 and 45 °C [97]. Additionally, Li et al. developed a new type of fluorescence temperature fiber optic sensor that uses NaYF_4_:Er^3+^, Yb^3+^ nanocrystals as the sensing unit. Results suggest that such UCNPs are highly stable and reliable, and prove the rationality of fluorescence fiber optic sensor’s design and its feasibility [98]. As a result, UCNPs are a great alternative for the design of temperature sensors, which account for the majority of the sensor market worldwide. It will promote the development of temperature sensors in industrial detection and other areas. Table 3 presents a summary of recent research on the development of UCNPs as attractive nanocarriers in biosensing applications.

### 5.3. UCNPs in Drug Delivery

The ability of nanocarriers to encapsulate poorly soluble drugs and minimize drug side effects is a significant advantage over traditional drug delivery systems. Nanocarriers are amenable for functionalization with imaging moieties and targeting ligands, further incrementing their functionality. UCNP-based drug delivery systems are reported to enhance the efficacy of a wide range of drug payloads and improve the solubility, stability, biodistribution, and pharmacokinetics of drugs [107,108]. Ln-doped UCNPs facilitate cell endocytosis and have good therapeutic effect due to their tiny particle size [109]. These nanoparticles subsequently release the drug and deliver them into tumor cells within a specified time limit. They improve the efficacy of the controlled drug release while reducing cell death, adverse effects, and tissue damage. UCNP-based nanocomposites are used as drug delivery systems and drug monitoring devices to address the needs of disease diagnostics and therapeutics. 

Photoinduced reactions such as photocleavage and photoisomerization are used in drug delivery systems. UCNPs, by virtue of their ability to emit UV light on excitation with NIR light, serve as attractive materials for photoinduced drug delivery. Photocleavage and photoisomerization of light-sensitive molecules can be induced by Yb^3+^ and Er^3+^ co-doped UCNPs and they can emit UV light under 980 nm NIR excitation, which is primarily used as probes for in vitro and in vivo bioimaging. UCNPs can enhance the efficiency of drug delivery when used to induce photocleavable reactions in presence of NIR light. Combination of the high penetration depth of NIR light and low energy requirement of UCNPs makes them compatible for use with economical CW lasers instead of high-energy pulsed lasers [110].

Wang et al. [111] prepared oleic-acid-capped β-NaGdF_4_: Yb^3+^, Er ^3+^ @β-NaGdF_4_ UCNP UCNPs coated with NIR light-absorbing polymer polydopamine (PDA) using a water-in-oil microemulsion method, to obtain monodisperse, stable, noncytotoxic core–shell-structured nanospheres UCNP@PDA core–shell nanocomposites. These hydrophobic NPs were subsequently modified with amino-terminated polyethylene glycol (mPEG-NH_2_) to improve the stability of UCNP@PDA in physiological conditions. The PDA shell exhibited a strong photothermal effect and provided an active surface for loading doxorubicin (DOX) via π–π stacking and hydrogen-bonding interactions. Owing to the high UCL emission, T1 relaxivity value, and CT contrast enhancement of UCNP cores, trimodal imaging (UCL/MRI/CT) of a mouse-bearing colorectal (SW620) tumor was achieved by PEGylated UCNP@PDA with a 5 nm thickness PDA shell (UCNP@PDA 5 -PEG). DOX-loaded UCNP@PDA 5 -PEG (UCNP@PDA 5 -PEG-DOX) demonstrated excellent synergistic efficacy in in vitro cell culture and in vivo animal experiments. Their results suggested that drug-loaded UCNP@PDA core–shell nanocomposite can be used as an efficient nanoplatform for biomedical applications, including chemo photothermal therapy and multimodality imaging. The UCNP@PDA 5 -PEG-DOX, in combination with 808 nm NIR-laser irradiation, exhibited a synergistic interaction between PTT and the enhanced chemotherapy, resulting in complete eradication of tumor without regrowth. The leakage study, hemolysis assay, histology analysis, and blood biochemistry assay revealed that these nanocomposites had negligible organ toxicity. These results indicate that UCNP@PDA 5 -PEG can be applied as efficient multimodality contrast agents for UCL, MR, and CT imaging. The advantages of this strategy include its simplicity as the dopamine monomers can be directly polymerized on the UCNP surface via water-in-oil microemulsion technique, and the thicknesses of PDA shell can be controlled by variation in the number of dopamine monomers used in the reaction mixture; its amenability to functionalization by reaction with thiol and amino-terminated molecules via Michael addition or Schiff base reaction; its utility as an efficient nanoprobe for UCL/MRI/CT multimodality imaging and the application of the PDA shell as a drug carrier with high photothermal conversion agent resulting in chemophotothermal synergistic killing of tumors. 

**Figure 8:**
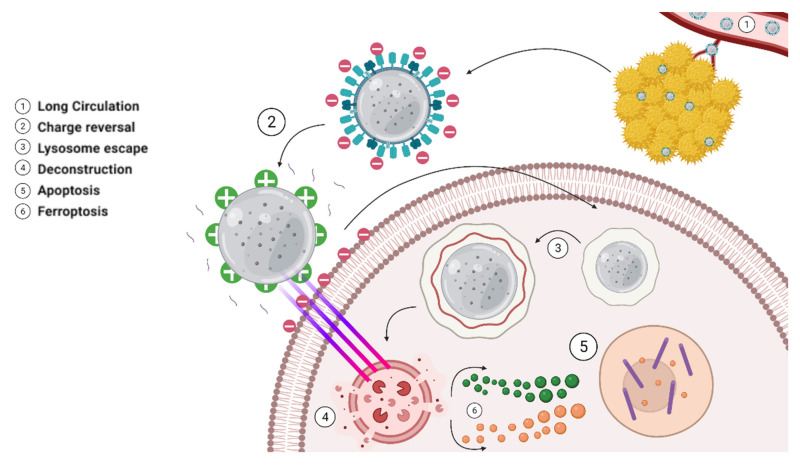
UCNP and DOX were loaded into gel nanoparticles and modified with PEI and DMMA to construct a nanolongan schematic with multiple transformations and corresponding anticancer mechanisms.

Liu et al. [112] developed a unique nanolongan delivery system that utilized a combination of ferroptosis−apoptosis (co-deliver an iron element and a chemotherapeutic drug) for improved anticancer efficacy of DOX. This comprised one core (UCNP) in one gel particle (Fe^3+^ cross-linked oxidized starch) with several on-demand conversions. The charge conversion of nanolongan surface sourced from 2,3-dimethyl maleic anhydride (DMMA) decoration conferred long circulation for utilizing the EPR effect and enabled more efficient uptake by tumor cells accompanied with subsequent lysosome escape. The core UCNP with the light conversion from NIR to UV circumvented the impediment of limited penetration depth and enabled the reduction of Fe^3+^ to Fe^2+^ (Figure 8). 

The nanolongan gel network developed could be deconstructed owing to this valence conversion, leading to the rapid release of Fe^2+^ and DOX. Cytotoxicity studies of the formulations developed (DGU: Fe; DGU: Fe + L; Dox; DGU: Fe/Dox and DGU: Fe/Dox + L) using the CCK8 assay on 4T1 and MCF7 cell lines indicated a dose-dependent response with DGU: Fe/Dox+ L formulation being the most cytotoxic, emphasizing the merits of co-operation of ferroptosis and apoptosis in the nanolongan formulations. Biodistribution studies in the 4T1-xenografted mouse model demonstrated that DMMA decoration resulted in prolongation of circulation time and increased tumor cell uptake via tumor-targeted delivery. In vivo efficacy studies in 4T1-xenograft mice indicated that DGU: Fe/Dox + L treatment showed enhanced therapeutic effect and complete tumor elimination at a higher dose, resulting in a 100% survival rate at 55 days along with a significant antimetastasis effect. Safety studies indicated that the DGU: Fe/Dox + L group showed lower toxicity than DOX due to targeted delivery to tumor. Their work demonstrated superior anticancer efficacy for this combination and can be extended to other anticancer agents for improved therapeutic efficacy. Table 4 presents a summary of recent research on the development of UCNPs as attractive nanocarriers in drug delivery.

### 5.4. UCNPs in Photodynamic Therapy (PDT)-Based Drug Delivery 

**Figure 9:**
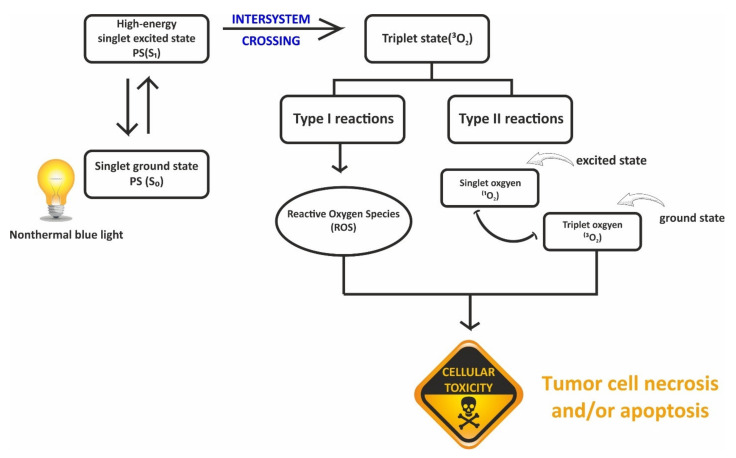
Type I and Type II reactions in PDT (photodynamic therapy).

PDT employs photodynamic effects to diagnose and treat several diseases including prostate cancer [121,122] and offers the advantage of low invasiveness and toxicity [123]. It involves three components, which include photosensitizer (PS) molecules, light source, and oxygen within the tissue at the disease site [124]. The principle of PDT showing the activation of the photosensitizer due to the excitation with light and the energy transfer to molecular oxygen is depicted in Figure 9. A light source is required for functioning in addition to the photosensitizers. Any light source with the appropriate wavelength and intensity can be utilized. Reactive oxygen species (ROS) created during PDT can eliminate tumors in a variety of ways, including directly triggering tumor cell necrosis and/or apoptosis, ultimately leading to cellular toxicity [125]. 

One of the most significant benefits of PDT is to treat the lesion area selectively under light irradiation while leaving normal tissues unaffected [126]. The engagement of UCNPs in PDT is clinically significant because it offers a novel way to treat deep-tissue tumors. It is based on the fact that UCNPs can efficiently convert deeply penetrating NIR light to visible wavelengths, which can activate photosensitizers, resulting in the production of cytotoxic ^1^O_2_. The PDT effect of the UCNP-based delivery system is evaluated by combining oleic acid-coated UCNPs with angiopep-2/cholesterol-conjugated poly (ethylene glycol) and hydrophobic photosensitizers [127]. The results showed that ANG-IMNPs could deliver dual photosensitizers to brain astrocytoma tumors selectively, resulting in successful PDT/PTT conjugation and a significantly enhanced median survival. The therapeutic efficacy of ANG-IMNPs demonstrated in this study implies that they can overcome the blood–brain barrier (BBB) and develop a glioblastoma multiforme (GBM) treatment. PDT research is undertaken to expand the platform of photosensitizers. It has the advantage of being able to provide the drug directly to the patient because cancer cells, due to their increased metabolism and absorption, only congregate in large numbers in tumors, causing no harm to healthy cells.

## 6. Pharmacokinetics (PK) of UCNPs

The absorption, distribution, metabolism, elimination, and toxicity (ADMET) together describe a drug’s overall disposition via pharmacokinetics, or what the body does to a drug, which plays a major role in drug development. The pharmacokinetic profile of UCNPs is an area with very few studies reported in comparison to the development of UCNPs for diverse biomedical applications. This is vital for assessing the feasibility of their translation to the clinic for therapeutic and diagnostic applications. Few toxicity studies of UCNPs developed as drug carriers and contrast agents are reported in mice with a vast number of UCNPs in vitro toxicity studies reported on different cell lines [128]. Most of the in vivo toxicity studies utilized the intravenous route of administration, although one study by Ortgies et al. reported toxicity studies of UCNPs developed for multiplexed imaging and drug delivery via the oral route of administration, which is the most common route for drug delivery.

Sun et al. [128] developed 50 nm-sized silica-coated NaYF_4_: Yb, Er NPs (NaYF_4_:Yb, Er@SiO_2_) and evaluated their bioavailability, biodistribution, and toxicity in mice via the oral and intravenous routes by using TEM and ICP–MS. Their results demonstrated that the biodistribution was a function of the route of administration, the UCNPs post-oral administration were absorbed in the intestine via Peyer’s patches as confirmed by TEM studies and the UCNPs via intravenous route were observed to be trapped in the hepatocytes. NaYF_4_: Yb, Er@SiO_2_ nanoparticles were found to accumulate in the bone, stomach, and intestine on oral administration, and in the liver and spleen on intravenous dosing. They did not report any significant toxicity of NaYF_4_: Yb, Er@SiO_2_ post 14 days oral dosing at 100 mg/kg. 

Li et al. [129] investigated the long-term in vivo distribution and toxicity of polyacrylic acid-coated NaYF4 upconversion nanophosphors (PAA-UCNPs) as NIR- to-NIR luminescence probes. The results of in vitro cytotoxicity studies showed that PAA-UCNPs had no significant effects on the proliferation of KB cells with retention of high cellular viability of more than 80% after 48 h incubation with PAA-UCNPs (480 mg/mL). Biodistribution studies illustrated that a high amount of PAA-UCNPs was found in the liver and spleen with a slow renal clearance. The results of in vivo toxicity studies in mice demonstrated that the mice survived for 115 days without any evident (observational, histological, hematological, and biochemical) toxic effects post intravenous dosing of 15 mg/kg of PAA-UCNPs. Their studies provide encouraging evidence in terms of safety and biodistribution for translating the use of PAA-UCNPs for long-term in vivo imaging to the clinic. 

## 7. Outlook and Perspectives

Upconversion nanoparticles represent a unique class of lanthanide-doped nanomaterials capable of converting near-infrared excitation into visible and ultraviolet emission. Their unique optical properties have expanded the landscape of their applications, ranging from drug delivery to bioimaging and biosensing. In this review, we presented current strategies used in the efficient synthesis of UCNPs and recent developments in their utility in biomedical applications. The intrinsic advantages of UCNPs, such as no background autofluorescence, minimal background tissue damage, and long penetration depth in biological tissues, are anticipated to keep UCNPs at the forefront of biological imaging and therapeutics. Their unique optical properties make them amenable to appropriate functionalization and they can be designed for effective delivery and release of drug payloads in response to a specific stimulus. Several NIR-responsive drug delivery systems based on UCNPs are reported in the literature, which appear to be promising additions in detection, bioimaging, drug delivery, and PDT, especially in cancer therapeutics. Some radiology departments have started using these modalities as the next-generation technology. There is scope for improving the efficiency of upconversion to promote wider utilization under in vivo settings and the development of surface treatment techniques for targeted drug delivery. 

Furthermore, the toxicity of these nanoparticles has lately been thoroughly addressed and the results of these investigations indicate that UCNPs show reduced toxicity in both in vitro and in vivo studies. Although various UCNP-based theranostic nanoplatforms have showcased immense potential, very few have entered into the clinical stage. Limited information on the long-term in vivo biological effects is one of the major barriers to the clinical translation of UCNPs. Given the complexity of many UCNP carrier systems, the question of the stability of these structures in the human biological environment for extended periods remains a biosafety concern. As a result, more research into the long-term stability of UCNPs in biological systems is required. Furthermore, once these UCNP-based nanoparticles are ingested, their function within the body and interaction with the nervous and immune systems is an important aspect that needs more explorative studies.

Another area that needs to be improved is the efficient integration of commercially available imaging equipment with upconversion nanocrystals. These nanocrystals, in contrast to fluorescent dyes and quantum dots, require excitation in the infrared regime, which means that standard commercial equipment cannot be used for quantitative measurements. Advances in fundamental nanophenomena in upconversion necessitate complex instrumentation, and a collaborative effort across a wide range of disciplines will have a significant impact in these areas. The detrimental effect of concentration quenching in luminescent materials restricts access to a high level of luminescence intensity, thereby limiting their future applications. The limitation imposed by the concentration quenching threshold becomes a real issue for nanoscale luminescent materials. 

UCNPs have attractive applications in biomedical and healthcare sectors including drug delivery, diagnostics, and theranostics. These applications depend on biocompatible products without or with negligible hazard to human cells, tissues, and organs. Translation of UCNPs is associated with challenges of reproducible synthesis, effective control over the NP size and shape, preparation of a biocompatible surface architecture with biorecognition elements for labeling target sites, light activation, theranostics, and drug delivery. Safety concerns and socioeconomic uncertainties are some of the important barriers to translational process in nanomedicine. Several deficiencies need to be addressed such as how they fit into clinical pathways, data consistency, and imaging techniques, but the potential is undeniable. With the present scenario of extensive research in nanotechnology development, it is conceivable that UCNPs will soon be placed among the mainstream nanoprobes that will be commonly utilized in both laboratories and clinics.

## Figures and Tables

**Table 1:** Methods for synthesis of UCNPs.

Methods	Conditions	Advantages	Disadvantages	Examples	References
Thermal decomposition	Higher temperature with an anhydrous anaerobic environment	High uniformity and monodisperse crystals, high luminous efficiency	Expensive, toxic byproducts are formed	NaYF_4_, NaYbF_4_, LiYF_4_	[44,45]
Co-precipitation method	Soluble salt solution, precipitant, coordinating ligand	Cost-effective with a simple operation process, ultrasmall UCNPs can be formed, usually requiring post treatment	Lack of particle size control	NaGdF_4_, LaF_3_, BaY_5_	[46,47]
Hydrothermal method	Higher temperature and pressure conditions	Simple and inexpensive, good control of morphology and the size of crystals can control the shape and size of the product	Nanocrystal growth process cannot be observed	NaYF_4_, NaYbF_4_, YVO_4_, BaYF_2_	[48,49]
Microemulsion method	Appropriate surfactant to stabilize a micelle and/or to control the growth of nanocrystals	Simple operation process, narrow size, high stability	In most cases, calcination or annealing is usually required	LaF_3_, NaYF_4_	[50]
Combustion method	Explosive reaction by heating, the reaction temperature is generally 500–3000 °C	Faster reaction time and less utilization of energy; controllable product quantity	Poor product purity and luminescence	Ba_5_(PO_4_)_3_OH: Er^3+/^Yb^3+^ Na_3_Y(PO_4_)_2_: Er^3+/^Yb^3+^	[51]
Sol-gel processing method	High luminescence intensity due to high crystallinity at high annealing temperature	Inexpensive precursors; small product size and simple procedures	Broad particle size and unsuitable for bioapplication	GdVO_4_	[52]

**Table 2:** Composition and synthesis of UCNPs for bioimaging application.

Composition/Modifier	Results	Route of Synthesis	References
β-NaY/GdF_4_: Yb, Er, Tm (UCNP)	Targets the lymphatic node, used for MR and CT imaging	Thermal decomposition	[81]
NaYF_4_:Yb^3+,^ Er^3+^ /DEVD peptide	In vitro and in vivo fluorescence results demonstrated the potency of tumor cell killing and significant suppression of tumor growth without any detectable side effects	Hydrothermal method	[82]
NaYF_4_:5%Nd@NaGdF_4_/DSPE-PEG2000	Strongest photoluminescence among the resultant NCs for NIR-II fluorescence imaging, and possess strong paramagnetism and X-ray attenuation for MRI and CT imaging	Liquid–solid-solution	[83]
NaLuF_4_:Gd^3+^/Yb^3+^/Tm^3+^/Oleic acid	Used for fluorescence imaging/MRI	Solvothermal method	[84]
NaYbF_4_:Tm^3+^/PEG	CT and strong NIR-fluorescent imaging that demonstrates both high in vitro and in vivo performances in the dual-bioimaging; very low cytotoxicity	User-friendly method [85]	[86]
NaYF_4_: Yb, Er@NaYF_4_: Yb, Nd UCNPs /Folate–chitosan	Effective UCL/CT imaging and combined chemotherapy and photothermal therapy	-	[87]

**Table 3:** UCNPs used in biosensing.

Mechanism	Biomarker	Probes	Limit of Detection (LOD)	Applications	Reference
Fluorescence	CaF_2_:RE^3+^@MSN+ Fe_3_O_4_	Oligonucleotide	100 nM	Multiple breast cancer-related miRNA biomarkers.	[99]
Fluorescence	Dipicolinic acid (DPA)	UCNPs−TPP/EBT	0.9 μM	Analysis of DPA in human serum.	[100]
Luminescence resonance energy transfer	Fe^3+^	NaYF_4_:Yb,Er,Tm@NaGdF_4_/ Nile red derivative (NRD) fluorescent	106.2 nM	Development of mPEG-UCNPs-NRD nanostructure used for detecting the intracellular Fe^3+^.	[101]
Fluorescence resonance energy transfer	Microrna-122	NaGdF_4_@NaGdF_4_: Yb,Er@DNA	10^−13^ M	Sandwich-hybridization observed between miR-122 and the designed DNAs.	[102]
Photoelectrochemical (PEC) aptasensing	Carcinoembryonic antigen	NaYF_4_:Yb, Tm@TiO_2_ upconversion microrods	3.6 pg/mL	NIR light-mediated PEC aptasensing system exhibiting a PEC response towards target CEA and its detection.	[103]
Fluorescence	Cyt c aptamer	NaYF_4_:Yb,Er@ NaGdF_4_@PDA@AP	20 nM	Intracellular Cyt c evaluation using UCNP@PDA@AP.	[104]
Luminescence resonance energy transfer	Carbohydrate antigen125 (CA125)	Polyacrylic acid (PAA)-coated UCNPs	9.0 × 10^−3^ U/mL^−1^	CA125 quantification in human serum, construction of point-of-care testing (POCT) devices.	[105]
Fluorescence	Prostate-specific antigen (PSA)	Anti-PSA antibodies	0.01 ng/mL	Biochip sensor for early diagnosis of cancer markers.	[106]

**Table 4:** UCNPs in drug delivery.

Material Composition	Payload Drug in UCNPs	UCL Excitation (nm)	Therapeutic Efficacy/Drug Loading Efficiency	Release Profile	Results	References
UCNPs@PDL *PDL-poly-D-lysine*	DOX	980 nm	-	-	Nanotheranostic agent developed to achieve highly localized therapy with great therapeutic efficacy against malignant tumors	[113]
NaYF_4_:Yb^3+^, Tm^3+^	DOX	980 nm	-	Increase in DOX release by activation of NIR light	Development of NIR light-triggered drug release of encapsulated DOX molecules by using UCNP/polymer nanomaterials in diblock copolymer self-assembly	[114]
UCNPs@MIL-PEG	DOX	980 nm	Therapeutic efficacy-60%	Less than 20% at pH = 7.4 UCNPs@MIL-100–60% after 30 h at pH 7.4 and 80% after 50 h; UCNPs@MIL-PEG reaches less than 20% at pH 7.4	Application of multifunctional UCNPs@MIL-PEG nanoparticles for UCL/MR dual-mode imaging and pH-responsive anticancer drug delivery	[115]
NaYF_4_: Tm^3+^, Yb^3+^	Nile Red	980 nm	-	-	Synthesized hybrid nanoparticles release the Nile red in response to a NIR-triggered drug release stimulus	[116]
NaYF_4_: Yb,Er/PAA/PEI nanoparticles	MDR1-siRNA	980 nm	Drug loading rate: 34.1%	50% MDR1-siRNA released from UCNP/PAA/PEI/MDR1-siRNA complex	UCNP nano complex—effective in gene silencing in paclitaxel-resistant ovarian cancer cells and resensitizes them to paclitaxel treatment	[117]
UCNPs@SiO_2_@PNBAM/MAA	DOX	980 nm	Drug loading rate: 7.23 wt%	Release rate constants and the correlation coefficients 4.15 × 10^−6^ and 0.98 (pH 7.4 and visible light), 2.64 × 10^−5^ and 0.99 (NIR light), 3.26 × 10^−5^ and 0.97 (pH 4.5 and visible light), 2.59 × 10^−4^ and 0.99 (pH 4.5 and NIR light), respectively	NIR irradiation and acidic conditions are beneficial to drug release; this controlled release feature makes the nanocomposite a promising carrier of drugs	[118]
NaYF_4_:Er/Yb@NaGdF_4_ ePEG	DOX	980 nm	-	-	Nuclear-targeted UCNPs-based theranostic systems combined with MR/optical imaging for cell nuclei and direct nuclear drug delivery functionalities to deliver drugs into the cell nuclei more efficiently	[119]
NaYF_4_:Yb/Tm/Er	hydrophobic AB3	980 nm	Loading efficiency: 16.7 wt%	Released without the 980 nm laser (<14 wt%) after 16 h. With a 10 min irradiation of 980 nm laser—nearly 75 wt% of drugs released after 16 h	A superior chemotherapy efficacy, whereas in vivo studies demonstrated that AB3-loaded UCNP-based micelles capable of targeted combination chemotherapy and PDT—provides a better antitumor efficacy compared to chemotherapy or PDT alone, without any apparent systemic toxicity	[120]
